# Soil ecotoxicology: state of the art and future directions

**DOI:** 10.3897/zookeys.176.2275

**Published:** 2012-03-20

**Authors:** Cornelis A.M. van Gestel

**Affiliations:** 1 Department of Ecological Science, Faculty of Earth and Life Sciences, VU University, De Boelelaan 1085, 1081 HV Amsterdam, The Netherlands

**Keywords:** Toxicity tests, bioavailability, ecological effects, biomarkers, soil organisms, isopods

## Abstract

Developments in soil ecotoxicology started with observations on pesticide effects on soil invertebrates in the 1960s. To support the risk assessment of chemicals, in the 1980s and 1990s development of toxicity tests was the main issue, including single species tests and also more realistic test systems like model ecosystems and field tests focusing on structural and functional endpoints. In the mean time, awareness grew about issues like bioavailability and routes of exposure, while biochemical endpoints (biomarkers) were proposed as sensitive and potential early-warning tools. In recent years, interactions between different chemicals (mixture toxicity) and between chemical and other stressors attracted scientific interest. With the development of molecular biology, omics tools are gaining increasing interest, while the ecological relevance of exposure and effects is translating into concepts like (chemical) stress ecology, ecological vulnerability and trait-based approaches. This contribution addresses historical developments and focuses on current issues in soil ecotoxicology. It is concluded that soil ecotoxicological risk assessment would benefit from extending the available battery of toxicity tests by including e.g. isopods, by paying more attention to exposure, bioavailability and toxicokinetics, and by developing more insight into the ecology of soil organisms to support better understanding of exposure and long-term consequences of chemical exposure at the individual, population and community level. Ecotoxicogenomics tools may also be helpful in this, but will require considerable further research before they can be applied in the practice of soil ecotoxicological risk assessment.

## Introduction

Ecotoxicology studies the effects of chemicals on organisms in the environment, with the final aim to protect the structure and functioning of ecosystems. This aim generally is achieved by assessing effects on single species of selected test organisms and trying to extrapolate the obtained (no) effect concentrations to safe levels for populations and communities. In the ecotoxicological risk assessment of chemicals, such safe levels are then compared with predicted or measured exposure levels to assess the possible risk for exposed ecosystems.

This paper will give an overview of developments in soil ecotoxicology, focusing on soil invertebrates, starting with a historical overview. Based on that, the state-of-the-art of current soil ecotoxicology will be depicted. This is done by first describing the way soil ecotoxicological data are used in the risk assessment of new and existing chemicals or the assessment of the risks of soil contamination. Next, the development of soil toxicity tests is outlined, followed by considerations on the inclusion of bioavailability, and the use of multiple species, model ecosystem and field tests. Then tools of assessing the possible risk of contaminated soils are described. Finally, some thoughts are given on the future of soil ecotoxicology. As this paper was written on the basis of a presentation at an isopod meeting, special attention will be given to the use of isopods in soil ecotoxicological testing.

## Historical perspective of soil ecotoxicology

When thinking of ecotoxicological effects, it is often referred to Rachel Carson, publishing her book ‘Silent Spring’ in 1962. This book was among the first describing the negative side-effects of the increasing use of synthetic pesticides that started from the second World war onwards. The book mainly focused on pesticide effects on birds, especially singing birds that apparently became silent due to the effects of chlorinated pesticides accumulating in the food chain. This book however, was not unique in ringing the alarm bell, although other bells did not sound that loud.

The first soil ecotoxicological papers date back to the 1960s, reporting observations on the negative effects of pesticides on soil invertebrates (e.g., Fox 1964, Edwards 1969). Similar to developments in aquatic ecotoxicology, these observations triggered the performance of toxicity tests with selected organisms to enable prediction of such side-effects in the field. First results of such toxicity tests, using Collembola and earthworms, were published by the end of the 1960s, also on pesticides (e.g., Ghabbour and Imman 1967, Scopes and Liechtenstein 1967). In the mean time, the Organization for Economic Co-operation and Development (OECD) started developing Guidelines for the testing of chemicals, to support the chemical risk assessment and pesticide registration procedures developed in most Western countries. It took another 15 years before the first toxicity test with soil invertebrates was internationally standardized by OECD, using earthworms and only focusing on short-term (acute) responses like survival (OECD 1984). In the 1980s and 1990s, the development of soil ecotoxicological tests received more attention, e.g. in the SECOFASE project funded by the European Union, that explored the possibilities of developing toxicity tests with different soil invertebrates, including earthworms, enchytraeids, nematodes, Collembola, staphylinid beetles, mites, centipedes, millipedes and isopods (Løkke and Van Gestel 1998). Several of the methods developed in SECOFASE never made it to standardization, but the project laid a basis for testing new species, using sub-lethal endpoints and including interactions between species.

During the last ten years, there has been a renewed attention for effects of *mixtures* of chemicals in soil (Van Gestel et al. 2011), while the interaction of chemicals with *other stress factors*, like temperature and soil moisture content, also came into focus (Holmstrup et al. 2010). In addition, the available test methods outline below are nowadays applied to new and *emerging chemicals*, especially to determine the toxicity of nanoparticles using earthworms (e.g. Shoults-Wilson et al. 2011a,b; Heckmann et al. 2011; Hooper et al. 2011), Collembola (Kool et al. 2011), and isopods (e.g. Jemec et al. 2008; Drobne et al. 2009; Pipan-Tkalec et al. 2010).

## Ecotoxicological risk assessment

In ecotoxicological risk assessment, two approaches can be distinguished. One approach aims at predicting possible effects of (new) chemicals in order to regulate their use or prevent their introduction onto the market. This predictive approach (*prognosis*) uses laboratory toxicity tests to obtain toxicity data that are used to derive safe levels of chemicals in the environment. The second approach is assessing the actual ecological risk or damage in case of pollution. This diagnostic approach (*diagnosis*) enables setting priorities for remediation and risk reduction, and may provide triggers for the management of contaminated land.

*Prognosis* starts from the paradigms also used in human toxicology. It assumes that the risk of a chemical for ecosystems can be estimated from its toxicity to a number of surrogate test or indicator species, exposed in standard laboratory toxicity tests. These tests aim at assessing toxicity, which is expressed in terms of dose-response relationships for effects on selected endpoints like survival, growth and reproduction. Toxicity is quantified by parameters like LC_10_ and LC_50_ (the concentrations killing 10% and 50% of the exposed test organisms, respectively), EC_10_ and EC_50_ (the concentrations causing 10% and 50% reduction, respectively in a measured endpoint, e.g. growth or number of juveniles produced), and NOEC and LOEC (no-observable and lowest-observable effect concentration, respectively). Since there is no ‘most sensitive species’ a battery of toxicity tests is needed to obtain proper insight into the potential hazard of a chemical for the ecosystem. In prognosis, the outcome of toxicity tests is used to establish thresholds or safe levels of chemicals in soil, which can be compared with measured or predicted exposure data (soil concentrations) to assess the (potential) risk.

A critical part of this procedure is the derivation of safe levels of chemicals on the basis of available toxicity data. When only short-term (acute) toxicity data are available (usually focusing on survival) or data for a limited number of species, somewhat arbitrary application factors are applied to derive safe levels that should protect ecosystems. For example, when only one or two LC_50_ values are available, a factor of 1000 is applied to the lowest LC_50_ value; this factor should be sufficient to extrapolate from acute to chronic effects (factor of 10), from one or few species to many species (factor of 10), and from laboratory to field (factor of 10). When sublethal toxicity data (usually NOEC or EC_10_ values for effects on e.g. reproduction) are available for 3 or more species, application of a factor of 10 to the lowest value is considered sufficiently protective. When many toxicity data are available (preferably ≥ 8) for species representative of different taxonomic groups (see below), a statistical method may be applied. Such a statistical method is used to construct a species-sensitivity distribution (SSD), which assumes a log-normal or log-logistic distribution of the sensitivities of species in an ecosystem. From such an SSD the 95% lower limit is selected as the safe level. At this level, at least 95% of the species in the ecosystem are supposed to be safe (Posthuma et al. 2002).

*Diagnosis* may use the same tools as applied for prognoses, but it more heavily relies on ecological methods and environmental chemistry. Basically, toxicity tests or bioassays are used as diagnosis tools to assess toxicity of soil samples from a contaminated site. Results of the bioassays, together with those of chemical measurements and ecological field observations, are used to assess the potential risk of soil contamination. The three tools together form the TRIAD approach (Jensen and Mesman 2006).

## Toxicity tests

Both prognosis and diagnosis use toxicity tests, and in both cases a battery of tests is recommended. Criteria to select tests for such a battery have been formulated e.g. by Van Gestel et al. (1997). These criteria among others include:

1. Practicability, referring to the feasibility and cost-effectiveness of a test;

2. Acceptability, including aspects like standardization, reproducibility and statistical validity of a test method as well as its broad chemical responsiveness;

3. Ecological meaning, including sensitivity and ecological realism of the test method.

To obtain a balanced battery of tests, in addition the following criteria need to be taken into account (Van Gestel et al. 1997):

1. Representativeness for the ecosystem to protect: this includes e.g. the representation of organisms having different life-histories, representing different functional groups, different taxonomic groups and different routes of exposure;

2. Representativeness of responses, to make sure responses measured really are relevant for the protection of populations and communities;

3. Uniformity, which refers to the possibility to apply all tests in a battery to the same test media.

By the end of the 1990s and early 2000 toxicity tests, using sub-lethal endpoints like reproduction, were standardized for enchytraeids, earthworms and Collembola by both the OECD and the International Standardization Organization (ISO). But also Environment Canada, the United States Environmental Protection Agency (EPA) and ASTM International (formerly known as the American Society for Testing and Materials) have described similar methods. Recently, for the same organisms, avoidance behaviour tests have been described, while for earthworms and enchytraeids a bioaccumulation test is available. Table 1 provides an overview of the toxicity tests with soil invertebrates available at the moment.

**Table 1.** A selection of available toxicity tests with soil invertebrates.

**Test organism**	**Species**	**Duration (days)**	**Endpoint**	**Guideline**	**Reference**
Earthworms	*Eisenia fetida*/ *Eisenia andrei*	14	Survival	OECD 207, ISO 11268-1	OECD (1984), ISO (1993)
		28 (+28)	Reproduction	ISO 11268-2, OECD 222	ISO (1998), OECD (2004b)
		2	Avoidance	ISO 17512-1	ISO (2008a)
	Field test, different species	Up to 1 year	Species diversity; abundance	ISO 11268-3	ISO (1999b)
Enchytraeids	*Enchytraeus albidus*, other *Enchytraeus* species	21 (+21)	Survival, Reproduction	ISO 16387, OECD 220	ISO (2004), OECD (2004a)
		2	Avoidance	No standard guidelines	Amorim et al. (2008a,b)
Mollusca	*Helix aspersa*	28	Survival, Growth	ISO 15952	ISO (2006)
Mites	*Hypoaspis aculeifer*	14	Survival, Reproduction	OECD 226	OECD (2008)
	*Platynothrus peltifer*	14, 70	Survival, Reproduction	No standard guideline	Van Gestel and Doornekamp (1998)
	*Oppia nitens*	28	Reproduction	No standard guideline	Princz et al. (2010)
		2	Avoidance	No standard guideline	Owojori et al. (2011)
Isopods	*Porcellio scaber*	28	Survival, growth	No standard guideline	Hornung et al. (1998a,b)
	*Porcellionides pruinosis*	14	Survival, reproduction	No standard guidelines	Jänsch et al. (2005)
		2	Avoidance	No standard guidelines	Loureiro et al. (2005)
Collembola	*Folsomia candida*, *Folsomia fimetaria*	28	Survival, Reproduction	ISO 11267, OECD 232	ISO (1999a), OECD (2009)
		2	Avoidance	ISO 17512-2	ISO (2011)
Insects	*Oxythyrea funesta*	14	Survival	ISO 20963	ISO (2005)
Carabid beetles	*Pterostichus oblongopunctatus*; *Poecilus cupreus*	Different durations	Adult or larval survival; adult behaviour, respiration	No standard guidelines	Schrader et al. (1998); Bednarska et al. (2010)

The oldest standardized toxicity test guideline with soil invertebrates, OECD 207 (OECD 1984), describes two short-term toxicity tests, one using 14 days exposure in soil and the other one exposing the worms for 2 days to treated filter paper. Both methods use survival as the only endpoint. The test on filter paper is only rarely applied nowadays, as it does not have any relevance for exposure in soil. It may however, be a useful exposure method for the rapid screening of chemicals, assessing the uptake and/or biotransformation of chemicals or other types of mechanistic research.

Compared to survival, reproduction is a more relevant endpoint when translating effects to the population level. For that reason, an earthworm toxicity test focusing on reproduction has been developed (ISO 1998, OECD 2004b). Although focus is on reproduction, it is essential in this test to also include weight change of the earthworms, since there is evidence for a trade off between reproduction and growth, which may affect their response to toxicants (Van Gestel et al. 1992, Van Gestel et al. 1995). Like for earthworms, the tests with enchytraeids (ISO 2004, OECD 2004a) also focus on reproduction and survival as the endpoint, but for these organisms no separate short-term (acute) and sub-lethal tests were developed. All the reproduction toxicity tests available with oligochaetes typically have test durations of 21-28 days. In case of the earthworm test with *Eisenia fetida* or *Eisenia andrei* and the enchytraeid test with *Enchytraeus albidus*, this means exposing adult worms for 28 and 21 days, respectively, after which they are collected from the soil; the cocoons are incubated for another 28 or 21 days, respectively to enable determining the number of juveniles produced. Nowadays, *Enchytraeus crypticus* seems to be more commonly used for toxicity testing than *Enchytraeus albidus*. Since that species is smaller, adults are not removed from the soil and reproduction is determined after 28 days of exposure. Considering the shorter life cycle of *Enchytraeus crypticus* and its high reproductive output, limiting test duration to 21 days has recently been advocated (Van Gestel et al. 2011).

A standardized test with snails has been developed by ISO (2006), focusing on survival and growth of juveniles snails (*Helix aspersa aspersa*) after 28 days exposure.

Standardized toxicity tests with soil arthropods include the collembolan species *Folsomia candida* (ISO 1999a) and *Folsomia fimetaria* (OECD 2009), the predatory mite *Hypoaspis (Geolaelaps) aculeifer* (OECD 2008), and larvae of the insect *Oxythyrea funesta* (ISO 2005). The tests with the collembolans and the predatory mite typically focus on survival and reproduction after 28, 21 and 14 days exposure, respectively for the parthenogenetic *Folsomia candida*, the sexually reproducing *Folsomia fimetaria* and the predatory mite *Hypoaspis aculeifer*. The test with insect larvae only focuses on survival.

Tests with carabid beetles have been performed using adult *Pterostichus oblongopunctatus* or larvae of *Poecilus cupreus*, but these tests have not been standardized and use different life stages (larvae, adults), endpoints (survival, mobility, respiration) and test durations (from few weeks to several months) depending on the aims of the study (e.g. Schrader et al. 1998, Bednarska and Laskowski 2008, Bednarska et al. 2010).

Also the toxicity tests with the oribatid mites *Oppia nitens* and *Platynothrus peltifer* described in the literature (Van Gestel and Doornekamp 1998, Princz et al. 2010) are not yet standardized. These tests focus on survival and reproduction.

For assessing chemical toxicity to isopods, also no standard test guidelines are available. Nevertheless, isopods are used as test organisms, using different test durations, different routes of exposure (food, soil) and different endpoints. Drobne and Hopkin (1995) described a test exposing *Porcellio scaber* via food and determining effects of zinc on feeding rates. Hornung et al. (1998a) developed a draft test guideline, allowing for exposure both via food and in soil, also using *Porcellio scaber* as the test species. Several different endpoints have been proposed, but considering the difficulty of culturing *Porcellio scaber* in the laboratory survival, growth and food consumption rate so far have been used most frequently (see e.g. Odendaal and Reinecke 2004, Nolde et al. 2006, Kolar et al. 2010). Another interesting endpoint may be related to the composition of the gut microflora of isopods (Drobne et al. 2002). Nevertheless, isopod (*Porcellio scaber*) reproduction also has been proposed as a test endpoint (Hornung et al. 1998b). Successful reproduction isopod experiments have been performed, e.g. by Van Brummelen et al. (1996a), in a 48-week test with *Oniscus asellus*, applying dietary exposure to Polycyclic Aromatic Hydrocarbons (PAHs), and by Lemos et al. (2010) assessing the reproduction toxicity to *Porcellio scaber* of some chemicals with suspected endocrine-disrupting effects. Other isopod species, like *Porcellionides pruinosis*, are more easily cultured in the laboratory, and therefore may be more suitable for performing reproduction toxicity tests (e.g., Jänsch et al. 2005).

Recently, avoidance response was introduced as an easy, fast and sensitive endpoint. For some chemicals avoidance response may be as sensitive as reproduction, while for others it is at least as sensitive as survival. Great advantage of avoidance tests is that they are fast, with test durations of no more than 2 days. Standard test guidelines for avoidance tests have been developed for earthworms (ISO 2008a) and Collembola (ISO 2011), but similar tests have also been performed with enchytraeids (Amorim et al. 2008a,b, Novais et al. 2010), oribatid mites (Owojori et al. 2011) and isopods (Loureiro et al. 2005, Zidar et al. 2005).

In addition to these tests with soil invertebrates, ISO and OECD have also developed a number of toxicity tests with plants, which are important in soil ecosystems as primary producers. Also, several tests are available focusing on the effects of microbial communities or processes performed by microorganisms, like nitrification.

Considering the fact that for a proper risk assessment a battery of tests is desirable, it is important to consider the currently available test methods. The current set of available tests (Table 1) shows an underrepresentation of arthropods in comparison with their abundance in the field when compared with other species like Oligochaetes. And of the available or suggested tests with arthropods, only the one with Collembola has been standardized. Development and international standardization of more toxicity tests with representative arthropod species therefore is highly needed (see criteria for the selection of test species outlined above). The ecological relevance of isopods, their typical routes of exposure (soil, food) and life history characteristics, the possibility to determine different endpoint, and the fact that they have already been used for testing for more than 30 years, make them highly suitable test organisms. Standardization of toxicity tests with isopods therefore is highly recommended.

## Bioavailability

For reasons of standardization and to facilitate comparison of results, all standardized tests use a standard soil type: the so-called OECD artificial soil, first introduced in the earthworm acute toxicity test developed by OECD (1984) This artificial soil is composed by mixing readily available materials like sphagnum peat (10%), kaolin clay (20%) and quartz sand (70%); by adding some CaCO_3_ pH is adjusted to approx. 6.0. The properties of this soil resemble those of a sandy loam soil. Recently, within OECD the use of 5% peat has been advocated when testing pesticides, in order to increase the ‘worst case’ realism of the artificial test soil for low organic (agricultural) field soils. Also with the aim to increase realism, the SECOFASE project started using the natural LUFA 2.2 soil (Løkke and Van Gestel 1998). The LUFA soils are commercially available from the Landwirtschaftliche Untersuchungs und Forschungsanstalt (LUFA) in Speyer, Germany. Since that time, LUFA 2.2 standard soil seems to become more commonly used for toxicity tests with soil invertebrates, also because several test species like the collembolan *Folsomia candida* and the enchytraeid *Enchytraeus crypticus* seem to perform as good in this natural soil as they do in artificial soil.

The notion that soil type was important when determining the toxicity of chemicals went along with the increasing insight into the concept of bioavailability: only a fraction of the total amount of chemical in the soil is available for uptake by organisms and therefore of relevance for risk assessment. This was, for instance, demonstrated by Bradham et al. (2006), exposing the earthworm *Eisenia andrei* for 28 days to different soil types spiked with one concentration (2000 mg/kg) of lead (Pb). While in some soils all worms died, in other soils no mortality was seen and in the remainder of the soils only part of the earthworms died. This finding could be explained from the differences in soil properties, especially pH, clay and organic matter content that affected the availability of lead for the earthworms. The pore-water hypothesis or equilibrium partitioning theory was developed to enable linking toxicity of organic chemicals to the concentration available in the pore water (Van Gestel and Ma 1988, 1990). For metals, application of this approach turned out to be less straightforward because metal speciation in soils is much more complex (Van Gestel 1997) and many factors may affect metal bioavailability in soils (e.g., Allen 2002, Lanno et al. 2004). It nowadays is realized that bioavailability is not a static but rather a dynamic concept (Luoma and Rainbow 2005, Van Gestel 2008). This was demonstrated by Van Straalen et al. (2005), exposing isopods (*Porcellio scaber*) from a metal-contaminated and a non-polluted site to zinc via the food. Both groups showed the same EC_50_ expressed on the basis of zinc concentrations in the diet, but contrary to the expectation effects could not be explained from zinc concentrations in the animals. So, it seems that flux of zinc through the animals rather than total zinc concentration was determining their sensitivity.

These findings also suggest that when considering bioavailability, not only chemical partitioning of chemicals in the exposure medium (soil, food) and pore-water concentrations have to be considered. The biology of bioavailability also needs attention. One aspect of this is the way organisms deal with chemicals. For metals, internal compartmentalization has been shown to be an important aspect (Rainbow 2002), as it determines what fraction of the metal is present in the body in a metabolically available form. Most soil invertebrates have the capacity to sequester at least part of their metal burden in such a way that it does no longer pose a risk. Isopods use the hepatopancreas for a very efficient storage of excess metals (Hopkin and Martin 1982), while earthworms have their chloragogenous tissue that serves the same purpose (Morgan and Morgan 1998, Morgan et al. 1999). As a consequence, both isopods and earthworms show a huge capacity of storing metals like cadmium, which after uptake are hardly eliminated. Other soil invertebrates, like Collembola and beetles, use the midgut epithelium for metal storage. Upon moulting, also the midgut epithelium is renewed enabling these organisms to excrete excess metal (Hopkin 1989). Internal sequestration determines what fraction of the total metal burden in an organism may contribute to its toxicity or is available for trophic transfer to its predators (Vijver et al. 2004). This was for instance demonstrated by Crommentuijn et al. (1994), who found very high Lethal Body Concentrations for cadmium in isopods compared to other arthropods that could be attributed to the highly efficient storage of the metal in an inert form.

Another way organisms may deal with potentially toxic chemicals is by biotransformation. The process of biotransformation aims at making chemicals more hydrophilic and in this way facilitating their excretion. Isopods and Collembola have been shown to be extremely efficient in biotransforming organic chemicals like Polycyclic Aromatic Hydrocarbons (PAHs), which are excreted by these organisms with half lives of approximately 1 day (Van Brummelen and Van Straalen 1996, Howsam and Van Straalen 2003, Stroomberg et al. 2004). Earthworms seem less efficient in doing so. Possible consequence of this rapid biotransformation is that potentially toxic metabolites may be produced. This has been shown in isopods, with even DNA adducts being formed upon exposure to PAHs (Van Brummelen et al. 1996a). This may also lead to long-term damage and possible effects on subsequent generations as has been shown for phenanthrene in the collembolan *Folsomia candida* (Leon Paumen et al. 2008). As very little is known about such multi-generation effects, further research on the long-term effects of chemicals is urgently needed.

An important biological aspect that may affect the exposure of soil invertebrates to chemicals is their behaviour. Soil by definition is a heterogeneous environment. As a consequence, also the distribution of chemicals in soil is heterogeneous. Chemicals reaching soil by areal deposition for instance accumulate in the topsoil layer, leading to a depth-related concentration gradient as was shown for PAHs in forest soils (Van Brummelen et al. 1996,b). Depending on the habitat and mobility, organisms may be more or less exposed to chemicals present in the topsoil layer. Similarly, the effect of pesticides on earthworms was shown to be highly related to their mobility with epigeic and anecic species being much more vulnerable compared to endogeic species. Especially anecic species, like *Lumbricus terrestris*, which come to the soil surface to forage and mate at night, may experience a very high exposure shortly after pesticide spraying (Edwards and Brown 1982). Also in case of spatially heterogeneous soil pollution, behaviour may affect exposure, as was shown for earthworm exposure to copper in a heterogeneously polluted soil by Marinussen and Van der Zee (1996) In the latter study, knowledge of the uptake and elimination kinetics showed to be very helpful in predicting metal concentrations in the earthworms living in a heterogeneously polluted environment. Also in case of isopods, behaviour is an important factor determining exposure. Unfortunately, no research has been done on the chemical exposure of isopods and consequent effects in relation to their behaviour in the field.

## Multiple species, model ecosystem (microcosm) and field tests

All standardized toxicity tests with soil invertebrates focus on assessing the effects of chemicals on single species of organisms. To enable assessment of toxic effects in a more realistic setting, micro- or model ecosystems have been developed, ranging from artificially composed set-ups with a number of selected different species introduced in a well homogenized soil (e.g., Burrows and Edwards 2004) to intact soil columns extracted from the field and incubated in the laboratory (e.g., Knacker et al. 2004). Such model ecosystems or microcosms allow assessing effects at the community level, taking into account the interactions between species. Although basically considered single-species tests, earthworm and isopod tests focusing on decomposition or feeding activity in fact also are multispecies tests as in these tests also the interaction with microorganisms in the gut and in the soil or food are important. In addition, the endpoint (decomposition) has high ecological relevance for assessing potential effects on the functioning of the soil ecosystem (e.g. Hobbelen et al. 2006). The only field test available aims at assessing pesticide effects on earthworms (ISO 1999b), but can be combined with a decomposition or litter bag test (Römbke et al. 2003, OECD 2006, Dinter et al. 2008).

Since the introduction of the term Ecotoxicology, the question for “putting more eco into ecotoxicology” has been raised. Some authors even argued that ecotoxicology should not be seen as a sub-discipline of toxicology but rather as a case of *stress ecology* (Van Straalen 2003). This notion has triggered the focus on more ecologically relevant test designs, integrated approaches including responses at different levels of biological organization, and taking into account the normal operating range of parameters describing the structure and functioning of soil ecosystems.

Since early 2000, with the notion of stress ecology, more complex issues have been receiving attention, with ecological vulnerability, trait-based analysis and effects on functional endpoints (so-called *ecosystem services*) being key items (e.g., De Lange et al. 2009, Saad et al. 2011). The application of these trait-based approaches in soil ecotoxicology on one hand offers promising perspectives, on the other hand it also demonstrates an enormous lack of knowledge on the traits represented by different species and groups of soil invertebrates.

## Diagnosis

Many of the tests initially developed for assessing the toxicity of single chemicals are also used for assessing the toxicity of field samples. In addition to the tests mentioned above, a bioassay using the nematode *Caenorhabditis elegans* has been developed by ISO (2010) Such *bioassays* may be applied together with chemical analysis and field observations. The resulting TRIAD approach is a useful tool for the actual risk assessment of contaminated sites (Jensen and Mesman 2006). ISO (2008b) gives guidance on the choice of different bioassays, depending on the purpose of the risk assessment and taking into account aspects like land use.

Other diagnostic tools include effects at the biochemical level. Such *biomarkers* may act as a sensitive, early warning indicator of possible effects at higher levels of biological organization (Spurgeon et al. 2005), and also may provide information on the mode of action of a chemical (Kammenga et al. 2000). Biomarkers may be applied both to organisms captured from the field and to test organisms exposed to field samples under controlled laboratory conditions (see e.g. Van Gestel et al. 2009). Isopods may be used for such biomarker studies (Köhler et al. 1999, Stroomberg et al. 1999, Stanek et al. 2006, Drobne et al. 2008, Lemos et al. 2009), while also their potential of accumulating metals has been proposed as a suitable monitoring tool (‘woodlouse watch’ scheme) especially in metal-contaminated areas (Hopkin et al. 1993).

Spurgeon et al. (2005), comparing different biochemical endpoints, demonstrated that responses at the gene level were most sensitive. This notion also plays a role in the recent developments of genetic tools (genomics, proteomics and transcriptomics etc.), which has resulted in a vast extension of the ecotoxicological tool box. *Ecotoxicogenomics* nowadays is seen as a tool to enable better understanding of molecular mechanisms of action of chemicals (Snape et al. 2004), while it may provide insight into the way soil invertebrates are able to develop resistance to pollution, e.g. metal or pesticide tolerance (Van Straalen et al. 2011). Ecotoxicogenomics may also help unravelling the mechanisms by which (metal-based) nanoparticles affect organisms, as e.g. was determined for the nematode *Caenorhabditis elegans* (Roh et al. 2009, 2010). Ecotoxicogenomics may also open the way for developing new diagnostic tools for assessing possible effects of soil pollution (Van Straalen and Roelofs 2008, Nota et al. 2010). Some authors have advocated that ecotoxicogenomics may enable bridging the gap between genes and populations (Fedorenkova et al. 2010). It remains however, uncertain whether time is ready for such ‘from gene to population extrapolations’ (Van Straalen et al. 2010). In a recent review, Van Straalen and Feder (2012) discuss the possible use of environmental genomics in the ecotoxicological risk assessment of chemicals. Community and population genomics may provide insight into the species composition at different sites and the possible relationship with pollution. Genome scans may also provide information on genetic changes in specific species that have been exposed to contaminated soils over many generations. Gene expression profiling may provide information on toxicant-induced changes in gene expression. The meaning of these changes however, remains unclear as the linkage between gene expression (transcriptomics) and the functioning of the genes (proteomics) often is not straightforward. At the moment, gene expression analysis is applied to only few species for which the genome has more or less completely been described, like the nematode *Caenorhabditis elegans*, the springtail *Folsomia candida* and the earthworms *Lumbricus rubellus* and *Eisenia fetida*, thus limiting wider application. Information on background gene expression is lacking, hampering a proper interpretation of responses under stressed condition. Van Straalen and Feder (2012) therefore conclude that more research is needed before genomics tools can make a sound contribution to the risk assessment of chemicals.

## Outlook

Final aim of (soil) ecotoxicology is the understanding of the long-term effects of chemicals on ecosystems. As such, focus on long-term sub-lethal effects is essential, but it also requires detailed understanding of the processes of exposure, uptake, internal processing (metabolism, sequestration) and intoxication in individual organisms as well as the translation of effects to higher levels of biological organization. From the overview presented in this paper, it may have become clear that soil ecotoxicology has shown a tremendous development in the past 40 years. From the initial realization that chemicals may affect soil organisms, through the development of standardized toxicity tests and the use of soil chemistry to develop the concept of bioavailability, soil ecotoxicology has grown to a mature field of science. The incorporation of biochemical and omics tools on one hand and the link with ecology on the other hand, does guarantee that soil ecotoxicology remains an important player in the field of stress ecology. In spite of the promising developments outlined above, the following aspects need further attention in the near future:

### Toxicity tests

Although several toxicity tests are available for soil organisms (Table 1), it is obvious that the current battery is not complete and also not well balanced. As mentioned above, it seems there is an under-representation of arthropods. Isopod toxicity testing seems most advanced, while these organisms also represent an ecologically important and relevant group of soil arthropods. In addition, they offer the possibility of exposure via soil and food, while effects may be determined at different levels including biochemical and genomics, individual (growth, behaviour) as well as ecological (feeding activity). It therefore is recommended to put more effort on standardizing isopod toxicity tests for sublethal endpoints. Finally, it has to be noted that the currently available toxicity tests may need adjustment to make them applicable for determining the toxicity of new and emerging chemicals, like nanoparticles.

### Bioavailability

For better enabling extrapolation from laboratory tests to the field and among soil types, it is essential to get better understanding of the routes of uptake of chemicals in organisms. This not only requires attention for the chemical aspects, but also needs a greater emphasis of the ‘biological’ aspects of bioavailability. This may also require paying closer attention to the way organisms are exposed in the field, and attention for the dynamics of exposure and bioavailability.

### Kinetics

For a better understanding of bioavailability but also of the toxicity of single chemicals and mixtures, it is essential to increase our understanding of toxicokinetics and toxicodynamics. Such understanding will also enhance the possibilities to extrapolate effects in time and to higher levels of biological organization, like the population level. Kinetics also is of great importance when considering the toxicity of new chemicals, like nanoparticles, that may show changing properties with time as a consequence of aggregation, agglomeration and dissolution processes. Finally, kinetics should not only address whole organisms but should also include the way organisms deal with chemicals internally (biotransformation, sequestration, internal distribution and translocation).

### Ecology

For better understanding exposure in the field and predicting ecosystem effects, our knowledge on the ecology of soil invertebrates needs much better development. Such knowledge also is crucial for the description of the normal operating range of structural and functional endpoints and for the application of trait-based approaches to understand and predict effects of chemicals on soil invertebrate communities and ecosystem services provided by these communities.

### Ecotoxicogenomics

For the application of genomics tools in the diagnosis of soil pollution it is essential to better understand the link between gene expression level responses and ecologically relevant endpoints. A better understanding of gene expression responses may also help unraveling the mechanisms of action of chemicals, single and in mixtures, and as such be helpful in predicting toxicity. In the long run, a better understanding of responses at the genomics level may even provide tools for cross-species extrapolation and the development of completely new models for mixture toxicity, especially when combined with toxicokinetics and toxicodynamics data. Genomics tools may also help unraveling the causes of long-term effects of chemicals, e.g. multi-generation effects as a consequence of accumulation of damage in earlier generations. But all these applications will require an enormous amount of information on the meaning of gene expression profiles in relation to background conditions, in relation to chemical exposure both outside and inside the body and related to ecologically relevant endpoints like growth and reproduction.
